# Mate choice for genetic compatibility in the house mouse

**DOI:** 10.1002/ece3.534

**Published:** 2013-03-20

**Authors:** Anna K Lindholm, Kerstin Musolf, Andrea Weidt, Barbara König

**Affiliations:** 1 Institute of Evolutionary Biology und Environmental Studies, University of ZurichWinterthurerstrasse 190, CH-8057, Zurich, Switzerland; 2 Department of Integrative Biology and Evolution, Konrad Lorenz Institute of Ethology, Veterinary University of Vienna and Austrian Academy of SciencesSavoyenstr. 1a, AT-1160, Vienna, Austria; 3 Zoological Institute, University of ZurichWinterthurerstrasse 190, CH-8057, Zurich, Switzerland

**Keywords:** Cryptic female choice, meiotic drive, segregation distortion, selection arena hypothesis, sexual selection, sperm selection, *t*-complex

## Abstract

In house mice, genetic compatibility is influenced by the *t* haplotype, a driving selfish genetic element with a recessive lethal allele, imposing fundamental costs on mate choice decisions. Here, we evaluate the cost of genetic incompatibility and its implication for mate choice in a wild house mice population. In laboratory reared mice, we detected no fertility (number of embryos) or fecundity (ability to conceive) costs of the *t*, and yet we found a high cost of genetic incompatibility: heterozygote crosses produced 40% smaller birth litter sizes because of prenatal mortality. Surprisingly, transmission of *t* in crosses using +/*t* males was influenced by female genotype, consistent with postcopulatory female choice for + sperm in +/*t* females. Analysis of paternity patterns in a wild population of house mice showed that +/*t* females were more likely than +/+ females to have offspring sired by +/+ males, and unlike +/+ females, paternity of their offspring was not influenced by +/*t* male frequency, further supporting mate choice for genetic compatibility. As the major histocompatibility complex (MHC) is physically linked to the *t*, we investigated whether females could potentially use variation at the MHC to identify male genotype at the sperm or individual level. A unique MHC haplotype is linked to the *t* haplotype. This MHC haplotype could allow the recognition of *t* and enable pre- and postcopulatory mate choice for genetic compatibility. Alternatively, the MHC itself could be the target of mate choice for genetic compatibility. We predict that mate choice for genetic compatibility will be difficult to find in many systems, as only weak fertilization biases were found despite an exceptionally high cost of genetic incompatibility.

## Introduction

The question of why females choose mates in the absence of any direct benefits, such as access to resources, protection from harassment and predators, or provision of parental care, is still not resolved. Much theoretical and empirical research has addressed the “good genes” hypothesis of mate choice, where females base mate choice on the quality of genes that their offspring would inherit through the sire (indirect benefits) (Andersson and Simmons 2006). More recently, the indirect benefits of “compatible genes” have been investigated (Mays and Hill 2004). Under the “compatible genes” hypothesis, a female bases mate choice decisions on the potential interaction between the genes inherited through herself and her mate. The difference can be thought of as mate choice for a mate's breeding value (good genes) versus for a beneficial combination of the genes of the parents (Puurtinen et al. 2005). Genetic compatibility has the potential to influence offspring quality as much as beneficial genes of the sire, and thus may strongly influence mate choice evolution (Neff and Pitcher 2005; Puurtinen et al. 2009). Selection for genetic compatibility requires an individual to reference its own genotype through self-inspection or familiar imprinting, as well as those of potential mates, and to choose mates accordingly. Whereas genetic compatibility is at the heart of conspecific mate preference, its role in mate choice within populations is not clear. At the population level, genetic compatibility is likely to be limited to specific genetic systems, because complex interactions of male and female genotypes across many genes would place severe constraints on any such system (Puurtinen et al. 2005). One such genetic system comprises the genes of the major histocompatibility complex (MHC), which are involved in immunocompetence and are believed to play an important role in mate choice (Jordan and Bruford 1998; Tregenza and Wedell 2000; Penn 2002; Milinski 2006; Yamazaki and Beauchamp 2007). It has been argued that genetic compatibility can only drive mate choice evolution within populations in two situations: inbreeding avoidance, in which the MHC has been implicated, and the coinheritance of compatibility and mate choice alleles (Tregenza and Wedell 2000).

**Figure 1:**
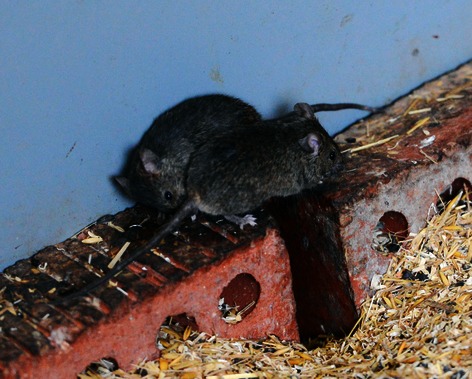
Picture of house mice in the free-living study population (by Sabine Wunderlin).

**Figure 2:**
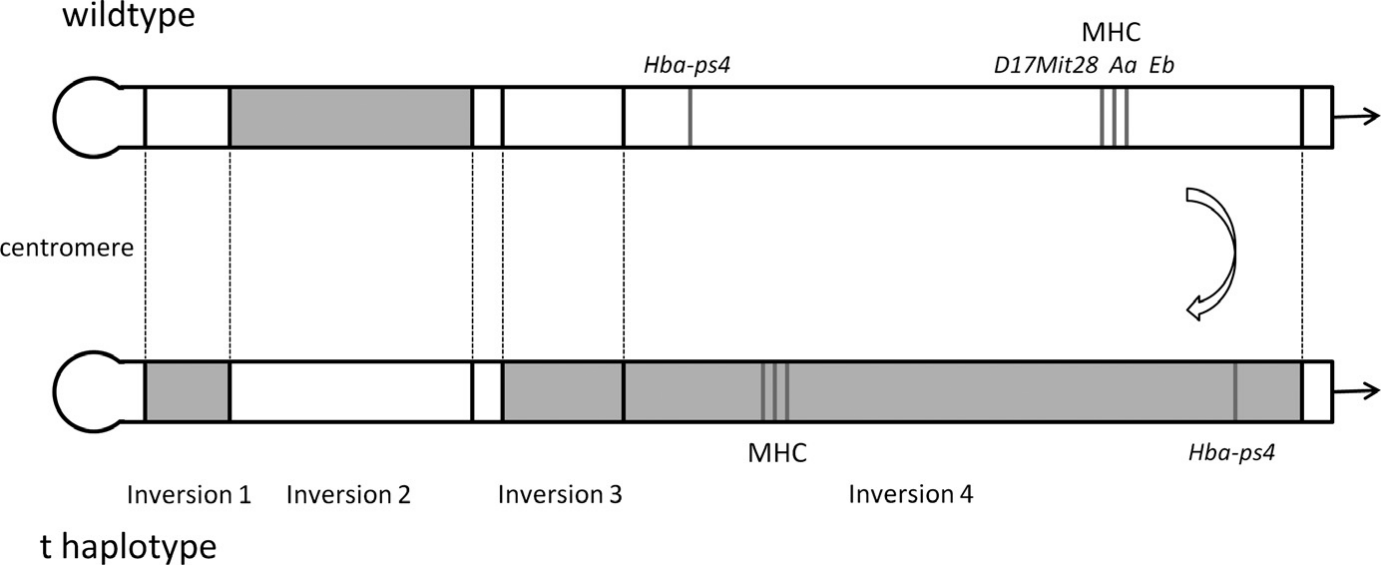
Schematic map of + and *t* haplotype forms of mouse chromosome 17. Shaded boxes represent the *t*-associated inversions. The MHC markers genotyped here are indicated. The *Hba-ps4* marker was used for *t*-haplotype identification.

Some of the strongest evidence for mate choice for genetic compatibility comes from the house mouse *Mus musculus* (Fig. 1) (Lenington and Coopersmith 1992), which carries a naturally occurring selfish genetic element, the *t* haplotype. The *t* haplotype evolved more than one million years ago and is composed of four linked inversions on chromosome 17 (Fig. 2) (Figueroa et al. 1985; Hammer et al. 1989), comprising a third of the chromosome (Silver 1993). The *t* haplotype contains at least one recessive lethal allele, and complementarity of recessive lethal alleles is used to define *t* haplotype variants (Artzt 1984). At least 16 different *t* haplotype variants are known (Klein et al. 1984). Heterozygotes of the same *t* haplotype variant produce *t*/*t* homozygotes that die before birth. However, heterozygotes for different *t* variants produce viable *t*/*t* offspring, but males are sterile (Dunn 1937). Moreover, the *t* haplotype shows drive (often called meiotic drive, segregation distortion, or transmission ratio distortion) in males (Chesley and Dunn 1936), which is the preferential transmission of one type of gamete to the next generation. Drive increases the proportion of offspring that inherit the *t*. To avoid *t*-related offspring mortality, heterozygous females should therefore prefer to mate with +/+ males. Such a preference would require tight linkage between the *t* and preference genes to avoid recombination breaking up the association (Price and Wedell 2008). Lenington and Egid proposed that a female preference gene lies within the *t* haplotype (Egid and Lenington 1985; Lenington and Egid 1985). They showed consistent odor preferences for +/+ males by +/*t* females and context-dependent preferences in +/+ females (Egid and Lenington 1985; Lenington and Egid 1985; Williams and Lenington 1993) in a series of experiments that have not been replicated elsewhere. It remains unclear if these laboratory-based odor preferences are generalizable to all *t* haplotype variants, and if they reflect actual fertilization bias when females mate in the wild, as female choice might be influenced by additional male quality traits (e.g., dominance status, MHC genotype, relatedness) and could be overridden by male mating behavior.

The *t* haplotype carries numerous MHC genes within its fourth inversion (Hammer et al. 1989). They code for cell receptors involved in presenting antigens to T-cells, thereby triggering specific immune responses against invading pathogens. Mice can smell the difference between congenic strains of laboratory mice that differ only at MHC alleles (Yamazaki et al. 1979), and in a laboratory setting, they choose mates according to MHC type (reviewed in Yamazaki and Beauchamp 2007). However, the extent to which MHC is used in individual recognition and mate choice in house mice is controversial (see Cheetham et al. 2007; Sherborne et al. 2007). The MHC could also provide a marker enabling postcopulatory (cryptic) female choice, via egg-sperm interactions (Wedekind et al. 1996), possibly modulated via MHC-linked olfactory receptor genes (Ziegler et al. 2002). If the *t* haplotype is associated with unique, but phylogenetically related, alleles at the MHC (e.g., Figueroa et al. 1985; Ben-Shlomo et al. 2007), MHC-mediated signals could provide the potential for female mice to discriminate between *t* and +.

In this study, we use a wild house mouse population and its laboratory-born descendants to address three aspects of mate choice for genetic compatibility. First, we use laboratory-born mice to estimate drive and empirically measure the cost of mating with a genetically incompatible mate in terms of litter size reduction. Costs to females could be low through overproduction of zygotes, leading to a litter size similar to that of genetically compatible crosses, despite prenatal mortality of *t*/*t* homozygotes (Charlesworth 1994), following from the selection arena hypothesis (Stearns 1987). Second, we use genetic paternity analysis to investigate whether wild females show a fertilization bias according to genetic compatibility. Finally, we address a mechanism that could allow females to discriminate between males on the basis of the *t* haplotype. We assess variation in the functionally important antigen binding sites of two MHC loci and a linked microsatellite to evaluate whether the *t* haplotype is associated with a unique set of MHC alleles.

## Methods

### Study species

Wild house mice (*Mus musculus domesticus*) were studied in a 72 m^2^ farm building near Illnau, Switzerland. This population was founded in November 2002 by 12 mice (four males, eight nonpregnant females) caught in the local area, from the two nearest known natural populations. Unknown to us at the time, mice from one of the populations were all +/+, while four of six mice released from the second population were +/*t*. The concrete floor was covered with sawdust and straw, 40 PVC nest boxes, and plastic tubes, bricks, and branches for use as hiding places. Vertical metal plates with holes to allow the passage of mice provided further substructure. Food (grains, oat flakes, and rodent pellets) and water were provided ad libitum at feeding and water stations. These conditions are similar to those found in natural house mouse populations, as house mice in Europe live commensally with humans and typically occur where food is plentiful, such as in stables or granaries (Berry et al. 2008). While the building was permeable to house mice through numerous small openings, larger predatory animals, such as cats, foxes, and owls were excluded, although these animals did occur outside the barn. For further details, see König and Lindholm (2012). All research described here received ethics approval and was conducted in accordance with Swiss law.

### *t* haplotype diagnosis

We identified *t* haplotype status on the basis of genetic tests of tissue samples (ear punches, and in some cases from the tail of deceased animals). DNA isolation was performed by salt-chloroform extraction (Müllenbach et al. 1989). For each animal we amplified the genotype at the *Hba-ps4* (alpha-globin pseudogene-4) locus, which occurs in the fourth inversion of the *t* haplotype, using the primers Hb.1 and Hb.2 (Schimenti and Hammer 1990). Compared to the + allele, the *t* allele at the *Hba-ps4* locus contains an insertion of 16 nucleotides, and this size difference is easily scored using a 3730xl DNA Analyzer (Applied Biosystems, Zug, Switzerland) and Genemapper software (Applied Biosystems). This method of scoring the *t* haplotype has been reliably used in previous studies (Schimenti and Hammer 1990; Huang et al. 2001; Carroll et al. 2004; Manser et al. 2011).

### Cost of genetic incompatibility

The cost of genetic incompatibility was assessed by comparing the number of offspring in laboratory crosses between virgin +/*t* females mated to +/*t* males or to +/+ males. As controls, we included the mating crosses of virgin +/+ females with +/*t* or +/+ males. This experiment was performed in an animal breeding facility of the University of Zurich under standard laboratory conditions in 2009. We used F1 to F3 descendants of wild-caught house mice from our Illnau study population caught between 2006 and 2008. All individuals were tissue sampled and genotyped at the *Hba-ps4* locus, as described above.

In the first experiment, we compared litter sizes at birth in 53 crosses between all combinations of +/+ and +/*t* males and females, avoiding sib–sib matings. An adult male and an adult female were placed together into a clean cage (Macrolon type III, 425 × 266 × 155 mm) with bedding (Lignocel® Bedding, London, U.K.) and nesting material (paper and cardboard). Food pellets (mouse and rat breeding diet from Provimi Kliba AG, Kaiseraugst, Switzerland) and water were provided ad libitum. After 14 days, males were removed. From 18 days postmating, females were checked daily for birth. If pups were found, or a female was no longer heavily pregnant, we searched the cage to locate all living pups as well as any that died after birth.

To investigate whether litter size differences were the result of genetic incompatibility leading to embryonic mortality, we conducted a second experiment using 74 crosses between all combinations of +/+ and +/*t* males and females, again avoiding sib–sib matings. Here, we estimated litter sizes at birth as well as prenatal mortality by examining placental scars in the mother's uterus shortly after birth. On the day of birth of the litter we euthanized the mother and her pups. Under a dissecting microscope, the uterus of each female was dissected and examined for uterine scars, as in Chesley and Dunn (1936). Two types of uterine marks were scored (see Fig. 2 of Krackow 1992). One type consisted of bloody markings in the middle of rosettes of tissue, referred to as “red” scars (Krackow 1990). The other type of mark consisted of yellow swellings, or “yellow” scars. Red scars indicate the implantation sites of pups which were just born, while yellow scars mark embryos that died after implantation and were resorbed (Krackow 1990, 1992). In this way we could estimate how many implanted embryos were viable versus inviable. One female was found to have only one functional uterine horn; she was then removed from the dataset. Data were analyzed in R 2.12.2 (R Development Core Team 2011), using a generalized linear model (GLM) in the MASS package (Venables and Ripley 2002) using litters as the unit of analysis and binomial errors. Contrasts were used for significance tests of differences between crosses. Pup survival from birth until weaning in the first experiment was analyzed similarly. Data were not overdispersed.

### Estimating drive

We estimated drive by examining the inheritance of the *t* haplotype in the breeding crosses above. The unit of analysis was the litter of a female, and the response variable was a vector comprised of the numbers of +/*t* and +/+ offspring in her litter. GLM modelling including confidence interval estimation was carried out using quasibinomial errors, using the MASS package in R 2.12.0. Our expected value for the proportion of +/*t* offspring at birth in heterozygote crosses was the expected number of +/*t* offspring divided by the expected number of +/*t* plus +/+ offspring or (0.5/(0.5 + [1 – TRD]/2)) (Manser et al. 2011), where TRD is the transmission ratio distortion estimated from crosses of +/*t* males mated to +/+ females.

### Mate choice for genetic compatibility in a wild population

#### Genetic parentage analysis

We performed a parentage analysis of our wild study population. The population was monitored regularly from November 2002 until February 2005 and from August 2005 until December 2005. For this study, we used all pups born (*N* = 201) between 27 July 2004 until 20 December 2005, as the male population was then relatively large, providing many males for females to choose between.

Nest boxes were checked every week for the presence of litters. Ear punches for genetic analyses were taken from pups at the approximate age of 13 days, before pups begin to be mobile. All mice were regularly captured (approximately once a month), sexed, and weighed. As adults, mice were again ear punched and implanted with Trovan transponders for individual identification using a portable transponder reader (LID 500 Hand-Held Reader, TROVAN electronic identification systems, Weilerswist, Germany). Dates of death of transpondered mice were recorded, and tissue samples for genetic analyses were taken from all untranspondered corpses that were found at the study site.

For parentage and identity analyses we used 21 microsatellite loci spread across 17 autosomes excluding chromosome 17, which carries the *t* haplotype. The loci (Chr1_20, D2Mit145, D3Mit278, D4Mit227, Chr5_20, D5Mit122, D6Mit139, D6Mit390, D7Mit17, D7Mit319, Chr8_3, D9Mit201, Chr10_11, D11Mit150, D11Mit90, Chr12_2, D13Mit88, D14Mit44, D16Mit139, D18Mit194, and Chr19_17) were amplified together with the *Hba-ps4* marker for *t* haplotype diagnosis, and a MHC-linked microsatellite (D17Mit28) in four multiplex polymerase chain reactions (PCRs). Marker details are available elsewhere (Meagher and Potts 1997; Bult et al. 2008; Teschke et al. 2008). PCR reactions used the Qiagen Multiplex PCR Kit or AmpliTaq Gold DNA Polymerase (Applied Biosystems) and a final concentration of 0.075–0.4 μmol/L primer for 28–31 cycles at an annealing temperature of 60°C. Negative and positive controls were included on each plate. PCR products were analyzed using a 3730xl DNA Analyzer and Genemapper software.

To test if variation at these 21 autosomal markers met expectations for neutral markers, we used a Hardy–Weinberg (HW) test as implemented in Genepop on the Web version 4.0.10 (Raymond and Rousset 1995; Rousset 2008), using a sample of all adult and subadult mice (25 females, 31 males) that were present in the barn in July and August 2004. There was no significant deviation from HW equilibrium in a global test across loci using Fisher's method (χ^2^ = 50.89, df = 42, *P* = 0.163).

Parentage analyses of the pup-mother-father trio were performed for 2004 and 2005 using cervus 3.0 (Kalinowski et al. 2007). Behavioral assignment of maternity is not possible in this population due to communal nesting. Parentage assignments were accepted at a confidence level of 95% with two or fewer mismatches between the mother–father–offspring trio. We considered as candidate parents all adult mice detected alive in the barn at least once within 30 days prior to the estimated birth date of a pup. While gestation in house mice lasts ca. 19 days (Theiler 1989), our 30 day period is conservative, but accounts for the possibility that individuals may be missed during individual monitoring sessions, and for variability in monitoring intensity. The genotyping error rate for cervus was determined by repeated PCR amplification and genotyping of 100 individuals, on average, per locus, for a total of 3837 alleles scored. This gave an error rate (frequency of alleles scored differently between PCR amplifications) of 0.006. An error rate of 0.01 was used in cervus analyses. In 2004, as monitoring was intense, we estimated the proportion of sampled mothers and fathers at 90%. The average number of candidate mothers per offspring was 21 and of candidate fathers was 24. The proportion of loci typed was 0.99. In 2005, with less frequent monitoring, we estimated the proportion of sampled mothers and fathers at 75%. The average number of candidate mothers per offspring was 42 and candidate fathers was 25. The proportion of loci typed was 0.98. Offspring without an assigned male and female parent were excluded from further analysis. Multiple paternity was scored when more than one sire was assigned to pups within a litter. The confidence interval around the multiple paternity estimate was calculated by bootstrapping the dataset, following the method of Eccard and Wolf (2009), which takes litter size into account.

#### Tests of the effect of genotype on paternity

Effect of female genotype at the *t* haplotype on paternity of offspring was assessed in several ways. In an initial analysis, we compared the proportion of offspring born to +/*t* and +/+ females sired by each genotype using Pearson χ^2^ tests. We then refined the dataset to singly sired litters to test female choice of sire for her litter, using Pearson χ^2^ tests. To allow for multiple paternity within a litter, we used a GLM approach. Two analyses were performed in R 2.12.0, both specifying a binomial error distribution. In the first, using the lme4 package (Bates et al. 2011), we used each litter as the unit of analysis. The response variable was comprised of two vectors: for each litter, the number of offspring sired by any +/*t* male and the number of offspring sired by any +/+ male. We used female genotype, proportion of +/*t* males present in the month before birth, their interaction, and year as fixed effects. Female identity was included as a random effect to account for repeated measures. The data appeared overdispersed. As a quasibinomial analysis using GLMM is no longer supported in lme4 (see http://cRAN.R-project.org/web/packages/lme4/ChangeLog), the significance of female genotype was (approximately) assessed by comparing deviance values of nested maximum likelihood models, including and excluding a predictor variable. Differences in deviance approximate a chi-squared distribution with one degree of freedom. We then considered a model in which each pup was considered an independent fertilization. Paternal genotype was the response variable, and we used the same fixed effects as above in a GLM analysis.

All analyses were performed using observed paternities, and where relevant, after applying a correction factor in the cases where a +/*t* female produced offspring from a +/*t* male. When such crosses occur, offspring of the *t*/*t* genotype will die prenatally and will not be sampled, thus underestimating paternity from +/*t* males. We accounted for this bias by multiplying the observed number of offspring per litter from +/*t* females and +/*t* males by the percentage of litter size reduction we observed in the laboratory in such crosses, and adding these as “virtual” pups to that litter. This had the effect of increasing the number of pups of *+*/*t* females sired by +/*t* males.

We also estimated drive from litters that were sired by males of a single genotype, analyzing them in the same way as the laboratory crosses (see above).

### MHC genotyping

We genotyped 29 mice from the barn population (15 +/+ and 14 +/*t* haplotype carriers, including all founder mice and a random sample of the population) at two MHC class II loci, Aα and Eβ on chromosome 17 (see Fig. 2) using single-stranded conformation polymorphism (SSCP). We amplified a fragment of exon 2 (antigen binding site) for both loci using the following primers: Aα-F: 5′-ACCATTGGTAGCTGGGGTG-3′ and Aα-R: 5′-CTAAATCCATCAGCCGACC-3′ for Aα (226 bp); JS1 5′-GAGTGTCATTTCTACAACGGGACG-3′ and JS2 5′-GATCTCATAGTTGTGTCTGCA-3′ for Eβ (171 bp) (modified after Schad et al. 2004). Ten microliter reactions contained 0.5–1 μL of extracted genomic DNA, 1 μl 10× Reaction buffer B (Solis BioDyne, Tartu, Estonia), 0.2 mmol/L dNTPs, 1.5 mmol/L MgCl_2_, 1U FIREPol® DNA Polymerase (Solis BioDyne) and 0.3 μmol/L of each primer for Aα, respectively, 0.5 μmol/L for Eβ. Cycling conditions consisted of an initial denaturation at 94°C for 2 min followed by 10 rounds of 30 sec denaturation at 94°C, 30 sec annealing at 59°C (Aα)/53°C (Eβ), and 60 sec extension at 72°C, followed by 25 rounds of denaturation at 94°C for 30 sec, annealing at 54°C (Aα)/48°C (Eβ) for 30 sec, 72°C extension for 60 sec. A final 10 min extension at 72°C followed the last cycle. For SSCP analyses, 1 μL of diluted PCR product (dilution Aα 1:60; Eβ 1:50) was combined with 14 μL loading dye mix (13.75 μL Hi-Di™ formamide, 0.25 μL GeneScan™ 350 ROX™ size standard [Applied Biosystems]). The mixture was denatured for 6 min at 95°C, immediately chilled on ice for 2 min and analyzed by capillary electrophoresis on an ABI PRISM® 3130xl automated DNA Sequencer (Applied Biosystems). The CE-SSCP polymer consisted of 5% conformational analysis polymer (CAP: made of 9% CAP, 10× genetic analyze buffer, 100% glycerol, and HPLC-water) and a 1× ABI running buffer was used. Separation of allelic variants was achieved by using the following run conditions: injection voltage at 1.2 kV, injection time of 18 sec, run voltage at 12 kV for 40 min, run temperature at 22°C. The retention times of the allelic variants were identified relative to the 350 ROX™ size standard using GeneMapper software.

Alleles were confirmed by direct sequencing of PCR products of ≥2 (preferentially) homozygote individuals following the manufacturer's instructions (Applied Biosystems). Sequences were edited and compiled with BioEdit 7.1.3.0 (Hall 1999). Sites involved in antigen binding were identified (Brown et al. 1993; Reche and Reinherz 2003). Microsatellite data obtained by paternity analyses (21 neutral markers) and one MHC-linked microsatellite (D17Mit28; Meagher and Potts 1997) were included for comparison of different selection patterns. We used the program Genepop (Raymond and Rousset 1995; Rousset 2008) to test for population differentiation and deviations from HW expectation.

## Results

### Cost of genetic incompatibility

The cost of genetic incompatibility is the reduction in litter size that a +/*t* incurs when mating with a +/*t* rather than a +/+. In experiment 1, we estimated the cost of genetic incompatibility to females by performing all possible crosses of +/*t* and +/+ genotypes and counting litter size at birth. Litter size differed between crosses (Table S1; ANOVA, *F*_3,49_ = 4.04, *P* = 0.012); +/*t* females mated to +/*t* males produced litters significantly smaller than those of all other crosses (for all contrasts, *t*_1,49_ > 2.34, *P* < 0.023). Litter sizes at birth may have been underestimated, as we found remains of dead pups in five instances, implicating infanticide, with a further suspicion of such causes in two more cases. To rule out maternal cannibalism, we conducted a second experiment, examining the uteri of females shortly after giving birth.

**Figure 3:**
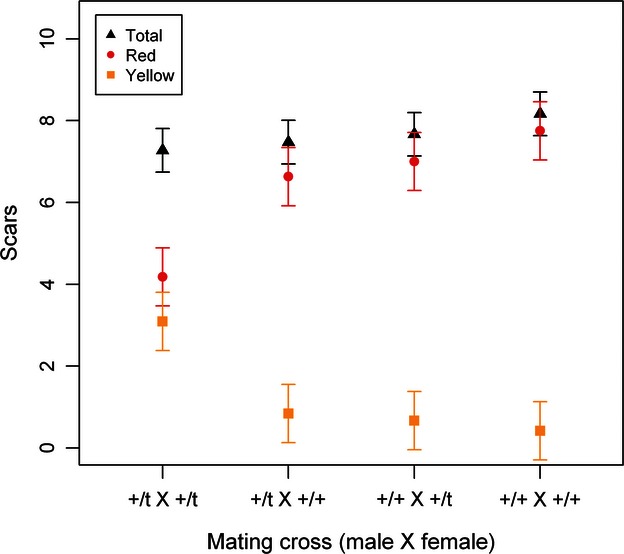
Number of uterine scars ± 95% CI per mating cross. Red scars indicate live births and yellow scars prenatal mortality.

In experiment 2, we conducted additional crosses to compare fertility, measured as the total number of uterine scars, which indicates the number of embryos implanted in the uterus. Fertility did not differ significantly among crosses (Fig. 3, ANOVA, *F*_3,70_ = 1.40, *P* = 0.249). The overall mean number of uterine scars per female was 7.58 (±0.15 SE). This indicates the average litter size at birth that would have resulted from the survival of all implanted embryos, regardless of genotype. Counts of red scars differed among crosses (Fig. 3, ANOVA, *F*_3,70_ = 16.12, *P* = 0.001). Red scars indicated that +/*t* × +/*t* matings resulted in a mean of 4.18 (±0.42 SE) offspring, significantly fewer than that detected in all other groups (*t*_1,70_ > 4.67 for all contrasts, *P* < 0.001), on average a loss of 3.40 pups per litter. Litter size at birth was highly correlated with the number of red scars (Pearson correlation, *N* = 74, *r* = 0.92, *P* < 0.001). Mean litter size at birth (Table S1) also differed significantly among the four types of crosses (ANOVA, *F*_3,70_ = 14.67, *P* < 0.001). While yellow scars were found in females of all mating crosses (Fig. 3), indicating prenatal embryonic mortality, there were significant differences between mating crosses (*F*_3,70_ = 16.73, *P* < 0.001). More yellow scars were found in +/*t* × +/*t* crosses, averaging 3.09 (±0.37 SE) (Fig. 3; *t* > 5.31 for all comparisons, *P* < 0.001). Compared with all other crosses combined, which averaged 0.67 (±0.16 SE), an excess of 2.42 yellow scars was found in +/*t* × +/*t* matings.

Overall, 79.3% of mating crosses were fecund (yielded offspring). Fecundity, however, did not differ according to type of mating cross (χ^2^_3,127_ = 1.85, *P* = 0.603). We also tested for a possible advantage to +/*t* females mated to +/*t* males – with a smaller litter size, they might give birth sooner. However, neither litter size nor mating cross predicted time to birth (*F*_4,119_ = 0.35, *P* = 0.842). Furthermore, using data from experiment 1, we tested for a difference in pup survival until weaning from different crosses and found no difference among crosses (binomial GLM, χ^2^_3,43_ = 0.16, *P* = 0.999).

The cost of genetic incompatibility to +/*t* females can be calculated by comparison of litter sizes at birth, of the number of red scars, or of yellow scars. Counting litter sizes after birth gave an estimation of reduction in litter size of 40.8% (combining experiments 1 and 2; 95% CI 30.6–50.8). Comparing red scars, which is a better indicator of the number of pups to which a female gave birth, the reduction is similar at 40.3%. Finally, from the excess of yellow scars in *+/t* × +/*t* matings compared to the average for all other matings, the reduction in litter size is estimated to be 32.0%. From the male +/*t* point of view, the cost of mating with a +/*t* female compared with a +/+ female was a litter size loss of 37.5% (combining experiments 1 and 2; 95% CI 26.8–48.2). From comparison of red scars, the reduction was 36.9%, while the estimate based on yellow scars is the same as for females.

### Drive

**Figure 4:**
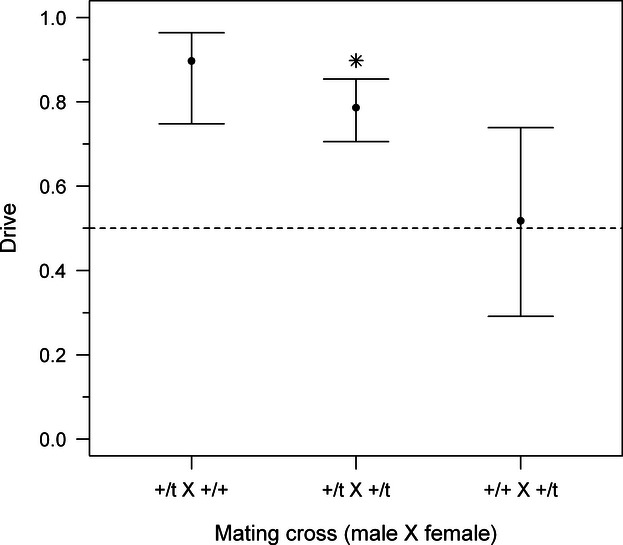
Drive estimates ± 95% CI for each mating cross. The expected value (asterisk) for crosses of +/*t* males and *+*/*t* females lies outside the observed value. The dashed line indicates the Mendelian expectation of 0.5.

Pups from the laboratory crosses were genotyped to estimate the degree of drive associated with the *t* haplotype. As expected, no pups were homozygous for *t*. Transmission ratios varied with the type of mating cross (GLM, χ^2^_2,91_ = 72.15, *P* < 0.001; Fig. 4). When +/*t* males and +/+ females were crossed, 89.7% of 175 offspring from 30 litters inherited the *t*. In crosses between +/*t* males and +/*t* females, 78.6% of 109 offspring that were born in 32 litters inherited the *t* haplotype, a significantly lower proportion than in the previous cross (GLM, *t* = 2.18, *P* = 0.032). In the reciprocal cross of +/+ male and +/*t* female, 51.8% of 203 offspring from 32 litters inherited the *t* haplotype, which was not different from 0.5 (exact binomial test, *P* = 0.674).

We further investigated the difference in transmission rate of the *t* haplotype inherited through the male, depending on the female genetic background. Based on 89.7% transmission to offspring in +/*t* male and +/+ female crosses, and 50% transmission in crosses of +/+ male and +/*t* females, the proportion of +/*t* offspring in crosses of +/*t* males and +/*t* females was expected to be 90.7%, taking into account *t*/*t* lethality. The 95% confidence interval around the estimate of the observed value (78.6%) does not overlap this expected value (Fig. 4).

### Mate choice for genetic compatibility in a wild population

#### Genetic parentage analyses and proportion of +/*t* males in the population

Both mother and father could be identified by genetic parentage analysis to 186/201 offspring at a confidence level of 95% (2004: 144/146 offspring; 2005: 42/55 offspring). Offspring where both parents could not be unambiguously identified were excluded from analysis: in 2004, two offspring could not be assigned fathers whereas in 2005, 11 offspring could not be assigned mothers and two additional offspring could not be assigned fathers.

**Figure 5:**
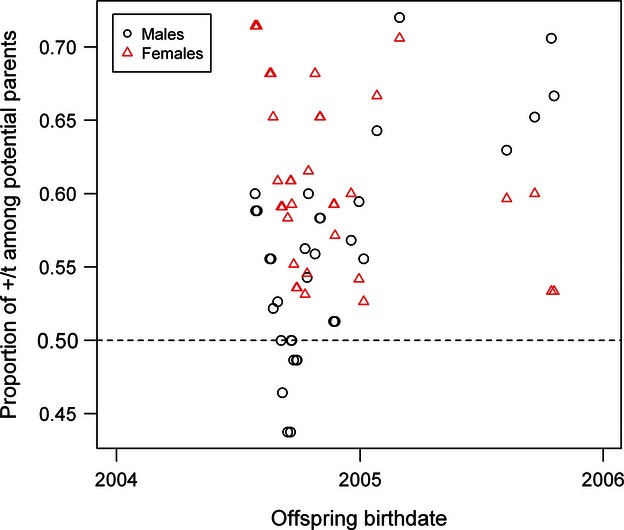
Proportion of +/*t* adult males and females according to litter birth date. The dashed line indicates a 1:1 proportion of +/*t* to +/+.

Frequency of the *t* among candidate parents at the time of putative mating, according to litter birth date, is shown in Figure 5. The proportion of males present at the time of putative mating that were of +/*t* genotype differed between 2004 and 2005 (Wilcoxon rank sum test, *W* = 144.50, *P* < 0.001), with an average proportion of 0.55 (±0.01 SE) in 2004 and 0.65 (±0.01 SE) in 2005. We therefore included a year effect in our analyses. These data are not truly independent, however, as individuals differed in tenure in the population. To test whether the actual proportions of +/*t* and +/+ males differed overall, we compared the genotypes of those individuals detected in the population (Table 1). Among the potential sires, the proportion of +/*t* did not differ from 0.5 (exact binomial test: 2004, 0.53 were +/*t*, *P* = 0.780; 2005, 0.59 were +/*t*, *P* = 0.200). Among potential mothers, the proportion that were +/*t* (0.63) differed from 0.5 in 2005 (Table 1; *P* = 0.023), but not in 2004 (*P* = 0.243). Although a higher proportion of +/*t* females (0.46) produced offspring in 2004 than did +/+ females (0.26), the difference was not significant (Pearson's χ^2^ = 1.18, df = 1, *P* = 0.277).

**Table 1:** Details of genetic parentage analyses

Genotype	*N* breeding females	*N* nonbreeding females	*N* breeding males	*N* nonbreeding males	*N* offspring	*N* offspring sired by +/*t*	Proportion of offspring sired by +/*t*	Lower SE–Upper SE	Corrected for *t*/*t* mortality
2004									
+/*t*	13	15	13	14	118	47	0.398	0.354–0.444	0.527
+/+	5	14	12	12	26	16	0.615	0.570–0.659	0.615
2005									
+/*t*	7	47	5	31	21	0	0	0–1	0
+/+	7	25	6	19	21	14	0.667	0–1	0.667
Combined									
+/*t*	19	49	17	25	139	47	0.338	0.299–0.379	0.462
+/+	10	31	16	16	47	30	0.638	0.595–0.678	0.638

#### Analysis of paternity patterns in relation to +/*t* genotype

Overall, 33.8% of pups of +/*t* females were sired by +/*t* males, compared to 63.8% of pups of +/+ females (Table 1), a highly significant difference (Pearson's χ^2^ = 11.83, df = 1, *P* < 0.001). However, the number of offspring sired by +/*t* males is likely to be underestimated for +/*t* females because of 40.3% prenatal mortality, our best estimate from the laboratory crosses (as *t*/*t* offspring die). If we correct for this, the estimate of proportion of offspring of +/*t* females sired by +/*t* males rises to 46.2%, which is still different from the proportion of offspring of +/+ females sired by +/*t* males (χ^2^ = 3.91, df = 1, *P* = 0.048). Multiple paternity in litters of two or more pups occurred in 32.5% (95% CI: 17.5–47.5) of litters overall. For +/*t* females, multiple paternity was observed in 9/24 litters in 2004 and 0/6 litters in 2005, or 30% overall (95% CI: 13.3–46.7). In four cases, paternity of the litter was divided between +/*t* and +/+ males. For +/+ females, 3/4 litters of two or more pups had multiple sires in 2004, and 1/6 in 2005, for a total of 40% (95% CI: 10.0–70.0). In two cases the paternity of the litter was shared by +/*t* and +/+ males.

Multiple paternity complicates analysis of female choice. We considered the simplest case, when a single male sired all offspring (i.e., no multiple paternity occurred), and asked if female genotype had an effect on her choice of sire. In 2004, 9/24 litters of +/*t* females and 2/4 litters of +/+ females were sired by +/*t* males. In 2005, 0/9 litters of +/*t* females and 4/6 litters of +/+ females were sired by +/*t* males. Overall, there was a significant effect of female genotype on paternity of the litter (Pearson's χ^2^ = 12.31, df = 4, *P* = 0.015). Data from 2005 alone showed a significant difference according to female genotype (χ^2^ = 5.13, df = 1, *P* = 0.023) but data from 2004 did not (χ^2^ = 0.01, df = 1, *P* = 0.937).

We then incorporated the possibility of having multiple sires within a litter into the analysis using a mixed-effect GLM with the proportion of pups in the litter that were sired by a male of +/*t* genotype, weighted by litter size, as the response variable. Predictor variables were female genotype, proportion of +/*t* males present before birth of the offspring, their interaction, and year. The dataset consisted of 56 litters from 29 females; therefore, we used maternal identity as a random effect to account for multiple litters from the same female. The interaction of female genotype and proportion of +/*t* males was significant (log-likelihood ratio test, χ^2^ = 9.56, df = 1, *P* = 0.002), as was the effect of year (χ^2^ = 27.70, df = 1, *P* < 0.001). Given the significant interaction term, the main effects marginal to it could not be tested independently (Fox 1997). If the interaction term was dropped from the model, then both female genotype (χ^2^ = 4.65, df = 1, *P* = 0.031) and proportion of +/*t* males (χ^2^ = 13.88, df = 1, *P* < 0.001) were significant. When data were corrected for *t*/*t* mortality, *P* values were yet smaller (not shown).

**Figure 6:**
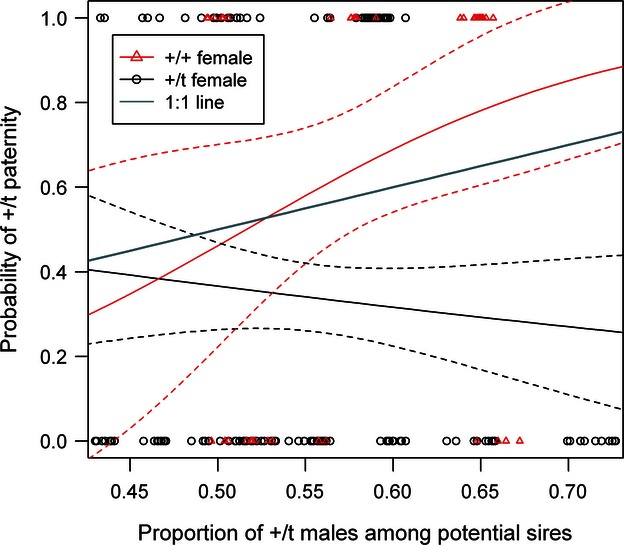
Incidence function of paternity by a +/*t* male relative to the proportion of +/*t* males among potential sires and to female genotype, with 95% confidence intervals. The gray line indicates a 1:1 relationship.

To further explore the interaction between female genotype and proportion of +/*t* males on sire paternity, we used a binary GLM with paternity of each pup as the unit of analysis. This assumes that paternity of pups within a litter is independent, which is not unreasonable as our data showed multiple paternity within litters. There was a significant interaction of female genotype with the proportion of +/*t* males in the population (χ^2^ = 15.85, df = 1, *P* < 0.001). +/+ females were more likely to have their offspring sired by +/*t* males when there were many *+/t* males available, while this had no influence on +/*t* females (Fig. 6). Female genotype was a significant predictor of sire genotype (χ^2^ = 12.94, df = 1, *P* < 0.001), while the proportion of +/*t* males in the population did not have a significant effect (χ^2^ = 0.00, df = 1, *P* = 0.961). Year also had a significant effect (χ^2^ = 14.55, df = 1, *P* < 0.001). Correcting for *t*/*t* mortality still gave significant (all *P* < 0.050) results for female genotype, its interaction with the proportion of +/*t* males, and year.

### Drive in the wild population

The degree of transmission bias of the *t* in litters from the wild population sired by a single genotype was estimated, and analyzed in a binary GLM, using litters as the main unit of analysis. The overall model was significant (*F*_2,42_ = 17.45, *P* = 0.003), which was due to a difference between crosses of +/*t* males mated with +/+ females, in which 24/28 (85.7%) offspring from seven litters inherited the *t*, compared to crosses of +/+ males mated with +/*t* females, in which 33/76 (43.4%) offspring from 25 litters inherited the *t* (*t*_1,42_ = 3.03, *P* = 0.004). The proportion of +/*t* offspring detected in these crosses did not differ from that of crosses of +/*t* males mated with +/*t* females, in which 26/40 (65.0%) of offspring in 13 litters inherited the *t* (GLM, *t*_1,42_ = 1.60, *P* = 0.117 for the former and *t*_1,42_ = −1.89, *P* = 0.066 for the latter). Transmission rate of the *t* was similar to the laboratory, for *+/t* × +/*t* crosses (Pearson's χ^2^ = 2.28, df = 1, *P* = 0.131), +/*t* males with +/+ females (χ^2^ = 0.09, df = 1, *P* = 0.761) and for +/+ males with +/*t* females (χ^2^ = 0.64, df = 1, *P* = 0.425).

### MHC genotyping

MHC class II loci Aα and Eβ and the MHC class I-linked microsatellite D17Mit28 showed similar levels of variation with 4–5 alleles per locus (summarized in Table 2). At each locus, +/*t* mice had the same allele which was expressed only as a heterozygote variant and was not found in any +/+ mice. Because the *t* haplotype is lethal in its homozygote form, heterozygote excess is anticipated in +/*t* mice. This was confirmed by significant heterozygote excess at the three MHC loci (Fisher's exact tests, *P* ≤ 0.004). +/+ mice exhibited a significant heterozygote deficiency at D17Mit 28 (Fisher's exact test, *P* = 0.001) and Eβ (Fisher's exact test, *P* = 0.005), but not at Aα (Fisher's exact test, *P* = 0.123).

**Table 2:** Allele frequency and heterozygosity of MHC loci among 14 +/*t* and 15 +/+ mice

	Locus					
	
	D17Mit28		Aα		Eβ	
			
Allele	+/+	*+/t*	+/+	*+/t*	+/+	*+/t*
**1 (*****t*****)**	**0.000**	**0.500**	**0.000**	**0.500**	**0.000**	**0.500**
2	0.233	0.036	0.167	0.036	0.067	0.107
3	0.067	0.107	0.067	0.000	0.233	0.036
4	0.367	0.250	0.768	0.464	0.700	0.357
5	0.333	0.107				
*H*_Obs_	0.400	1.000	0.267	1.000	0.200	1.000
*H*_Exp_	0.720	0.688	0.393	0.553	0.467	0.632
Exact tests						
*P*	<0.001		<0.001		<0.001	

Alleles are arbitrarily numbered; in bold are *t-*specific alleles. Exact tests for population differentiation are given (Raymond and Rousset 1995).

**Figure 7:**
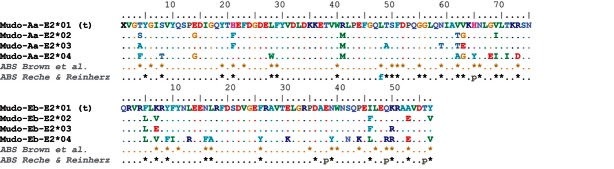
Amino acid sequence of MHC class II genes aligned: (A) Aα and (B) Eβ. Asterisks indicate sites involved in antigen binding identified from Brown et al. (1993) and Reche and Reinherz (2003); p = residues in proximity to the antigen but likely do not contribute to the specificity and binding properties of the molecule, f = residues interacting with flanking regions of the peptide core extending beyond the binding groove (Reche and Reinherz 2003).

Sequences of Aα and Eβ indicate that all alleles were unique not only with respect to nucleotide sequences but also at the amino acid level (Fig. 7). Sites involved in antigen binding showed the highest variation with an average of 5.7–7.2 differences in amino acid sequence between alleles compared with 1.8–2.0 differences at neutral sites, indicating that all alleles found might differ in their antigen binding capacities.

These mice were also genotyped for 21 neutral microsatellites. Similar to the pattern observed at the MHC loci, a significant deficit of heterozygotes was found but only for +/+ mice (Table 3, Fisher's exact test, *P* < 0.001) but the difference between observed and expected heterozygosity dropped to a fourth of the value found at MHC loci. To clarify the selective processes acting on the different loci we excluded the 12 founder individuals, and analyzed the remaining random sample of their descendants (*N* = 17). For MHC markers, the excess of heterozygotes in +/*t* remained significant, but a deviation from HW equilibrium was no longer observed in the +/+ population. Neutral markers showed the lowest differences between observed and expected heterozygosity in this sample with no deviation from HW equilibrium, however, a significant heterozygote excess was found in the +/*t* population (Table 3). Differentiation between the +/*t* and +/+ population was significant (Fisher's exact tests, *P* < 0.001) in all analyzed comparisons.

**Table 3:** Variability in three MHC loci and 21 neutral microsatellites for +/*t* and +/+ individuals, and divided into the subset of 12 founders and the subset of 17 randomly chosen individuals

Type of loci	*N* individuals	*N* loci	Mean *N* alleles/locus	*H*_Obs_	*H*_Exp_	*H*_Obs_–*H*_Exp_	*P*
MHC							
+/+	15	3	3.33	0.289	0.526	−0.238	0.001
*+/t*	14	3	4.0	1.000	0.624	0.376	0.000
MHC – excluding founders							
+/+	7	3	3.00	0.286	0.414	−0.128	0.080
*+/t*	10	3	3.33	1.000	0.605	0.394	0.000
Microsatellites							
+/+	15	21	5.29	0.643	0.728	−0.085	0.000
*+/t*	14	21	4.67	0.694	0.615	0.079	0.995
Microsatellites – excluding founders							
+/+	7	21	4.14	0.703	0.720	−0.017	0.955
*+/t*	10	21	4.29	0.705	0.631	0.073	0.999

## Discussion

Two features of the *t* haplotype are well known: it causes drive in males, and known variants of the *t* haplotype carry a recessive lethal allele. Together, these two phenomena result in a reduction in litter size when +/*t* females produce offspring sired by +/*t* males, a form of genetic incompatibility. The cost and consequences of genetic incompatibility have, however, rarely been measured. Here we have used a combination of laboratory experiments and field data from house mice to do so.

### Cost of genetic compatibility

For a +/*t* female or male, mating with a +/*t* rather than a +/+ partner resulted in a litter size reduction of 40%. This corresponds empirically to a reduction in litter size from seven pups to four pups. Examination of uterine scars showed that the reduction in litter size in +/*t* × +/*t* matings is the result of prenatal mortality, as fertility (the number of implanted embryos) did not differ between crosses. Thus, we found no evidence for reproductive compensation for the loss of homozygous offspring, as has been postulated (Charlesworth 1994). Fecundity, the ability to produce offspring, also did not differ between crosses. Our results are consistent with the presence of a recessive lethal allele within the *t* haplotype, but suggest no additional deleterious effects of the *t* on fertility and fecundity in monogamous matings. This is in contrast with previous studies, which showed reduced fertility in +/*t* male but not female mice (Lenington et al. 1994; Carroll et al. 2004) and reduced fecundity of +/*t* females (Carroll et al. 2004). However, selfish genetic elements that affect sperm are generally thought to be associated with a reduction in fertility (Price and Wedell 2008; Wedell 2013).

No mice were found to be homozygous for the *t* haplotype. Although populations are known which host multiple variants of the *t* (Petras 1967; Baker 2008), we found only a single *t* haplotype in our study population.

### Drive

Drive of the *t* haplotype amplifies the cost of genetic incompatibility by increasing the frequency at which *t*/*t* are produced when both males and females are carriers. In laboratory crosses between +/*t* and +/+, we found that the *t* haplotype when transmitted solely paternally is inherited by nearly all offspring (a proportion of 0.90), which is consistent with the range of 0.88–0.99 reported in crosses of wild mice (Dunn 1957; Ardlie and Silver 1996; Carroll et al. 2004). In contrast, through females the *t* haplotype was inherited by 0.52 of offspring, consistent with Mendelian expectations and similar to previously reported findings of 0.43–0.45 (Ardlie and Silver 1996; Carroll et al. 2004).

### Postcopulatory female choice

The proportion of offspring inheriting the *t* in heterozygous matings was significantly lower, at 0.79, than in matings between +/*t* males and +/+ females, at 0.90. This reduction in drive has fitness consequences, as more viable offspring will be produced, increasing litter size by 8.3%. Female genetic background thus appears to influence fertilization success of *t* sperm relative to + sperm within the same male. In the only previous study to have compared drive in males according to female *t* genotype within a strain, the proportion of offspring inheriting the *t* in heterozygous matings was 10% lower than in matings between +/*t* males and +/+ females (Bateman 1960). These two results suggest that in wild mice, the *t* haplotype in females modifies drive in males. Modifiers of drive in natural populations have not been previously found in the *t* haplotype system (Ardlie and Silver 1996), but are known from other selfish genetic elements (Montchamp-Moreau et al. 2001).

Fertilization bias as a result of egg-sperm interactions is a possible mechanism for the lower than expected transmission of the *t* haplotype. Within-male sperm competition is an unlikely mechanism for the fertilization bias, as the bias depends on female genotype. Eggs carrying the *t* will die if fertilized by a *t* sperm, thus all else being equal, selection will favor a mechanism whereby *t* eggs can recognize and avoid fertilization by *t* sperm. Sperm selection is well documented in some sessile hermaphroditic organisms, but there are few examples from other groups (Birkhead and Pizzari 2002, reviewed in Zeh and Zeh 1997). The effect detected in house mice was significant but weak, with an effect size of 10–11% (this study and Bateman 1960).

### Precopulatory female choice

Choice of mating partner, rather than choice of sperm, is another way to avoid the cost of fertilizing eggs with genetically incompatible sperm. In free-living house mice, we found that offspring paternity was nonrandom with respect to female and male genotype. +/*t* males sired 30.0% more offspring with +/+ than with +/*t* females. Even after accounting for the expected prenatal mortality of *t*/*t* pups, the fertilization bias remained (a difference of 17.6%). For +/+ females, higher frequencies of +/*t* males led to higher rates of paternity by +/*t* males, but this was not the case for +/*t* females. It is perhaps surprising that the difference in paternity of +/*t* and +/+ females is not stronger because of the cost of genetic compatibility to +/*t* females. Multiple factors can influence paternity outcome: mate choice for a variety of traits at the pre- or postcopulatory stage (e.g., preference for dominant males; Coopersmith and Lenington 1992; Rolland et al. 2003; Carroll et al. 2004), and constraints on females being able to exclusively mate with preferred males, for example because of risk of infanticide by territorial males (Vom Saal and Howard 1982). Environmental variation, such as family genotype, has been previously found to influence preferences of +/+ females, whereas preferences of +/*t* females for +/+ males persisted (Lenington 1991). Controlled laboratory experiments are needed to clarify our results. Nonetheless, this is the first evidence that +/*t* females avoid mating with genetically incompatible males in a wild population. Such field evidence is still lacking from other selfish genetic elements.

While we found no differences in postnatal pup mortality in our laboratory study, we cannot reject the possibility of mortality differences in the wild population, which could influence the results of our mate choice analysis and estimates of drive. However, postweaning survival in the wild population is similar between +/*t* and +/+ males, whereas +/*t* females outlive +/+ females (Manser et al. 2011).

### Sperm competition

Multiple mating by females could also contribute to a fertilization bias in favor of +/+ males (Haig and Bergstrom 1995), due to the mechanism responsible for drive of the *t* haplotype. Drive is the result of the actions of several loci within the *t* haplotype (Lyon 2003), which act during spermatogenesis when intercellular bridges link syncytial spermatocytes and spermatids (Dym and Fawcett 1971). Products of these loci hyperactivate the sperm mobility kinase allele of + sperm (Herrmann et al. 1999; Bauer et al. 2005, 2007), while *t* sperm are protected by haploid expression of the *t*-specific allele of the same gene (Véron et al. 2009). The disruption of gene regulation in + sperm leads to an impairment of + flagellar function. While this gives an advantage to *t* sperm relative to + sperm in within-ejaculate sperm competition, +/*t* males provide a smaller number of functional sperm relative to +/+ males and are thus at a disadvantage in between-male sperm competition, assuming a fair raffle mechanism (Parker 1998). Lower sperm counts of male mammals have been associated with reduced success in sperm competition (Stockley 1997; Preston et al. 2001). Thus, if +/*t* females did not discriminate between mating partners, but simply mated with multiple males, then there is strong theoretical support that a fertilization bias against +/*t* males would result (Haig and Bergstrom 1995; Manser et al. 2011). Such an effect has been demonstrated in *Drosophila pseudoobscura* that carry a selfish genetic element affecting male sperm (Price et al. 2010), a system in which females are not able to discriminate between carriers and non carriers (Price et al. 2012).

We found two lines of evidence that argue against the sperm competition hypothesis as the sole mechanism for fertilization bias in the context of our paternity analysis. First, our result that +/+ females were more likely to have their offspring sired by +/*t* males when +/*t* males were more abundant in the population, but +/*t* females were not, argues for random mate choice in +/+ females, but not in +/*t* females. Furthermore, when +/*t* males are present at high frequency, then multiple mating will be less effective in avoiding the costs of genetic compatibility. In this context, +/*t* female mate choice for +/+ males is of most benefit. Second, we did not find evidence for a high rate of multiple paternity, however, confidence in our estimates was low due to small sample sizes and small litter sizes. 32% of litters were sired by multiple fathers, and only 15% were sired by both a +/*t* and a +/+ male, which is the context in which sperm competition could produce a fertilization bias. This estimate of multiple paternity is similar to those observed in other studies of wild house mice (12–31% in Dean et al. 2006, 6–43% in Firman and Simmons 2008a), but is relatively low in comparison with other rodent species (Eccard and Wolf 2009). Additional studies are needed to test if multiple mating differs between female genotypes and to estimate the paternity share that results from multiple matings involving males of both genotypes. Moreover, it is important to note that molecular estimates of paternity provide data on male fertilization success, and the actual mating rate (including both successful and unsuccessful males) could be much higher.

### MHC – the mechanism underlying fertilization bias?

In summary, we found evidence for a fertilization bias acting in *+*/*t* females against the *t* at two different stages, one at the level of choice of sire and one at the postcopulatory stage, at the level of choice of sperm. How could such a bias arise? Through comparison of loci within the *t* haplotype, we showed that +/*t* mice carry unique alleles at functionally important genes of the MHC. In contrast, neutral microsatellite markers show no differences in allelic variants between +/*t* and +/+. This is consistent with reports of a unique MHC allele associated with the *t* haplotype in a study of Israeli house mice (Ben-Shlomo et al. 2007), and a study of H2 (MHC) antigens, which detected closely related MHC antigens in different strains of *t* haplotype mice (Figueroa et al. 1985).

The case that the MHC plays a role in fertilization bias is strengthened by evidence that it plays a role in postcopulatory sexual selection. House mouse sperm cells have been reported to express MHC antigens (Fellous and Dausset 1970; Martin-Villa et al. 1999; Ziegler et al. 2002) and olfactory receptor genes (Fukuda et al. 2004). *t* and + sperm and testicular cells have also been found to differ in expressed antigen (Yanagisawa et al. 1974; Cheng et al. 1983), but see (Gable et al. 1979; Goodfellow et al. 1979). Furthermore, female house mice of different genetic backgrounds differ in how quickly they transport sperm (Nicol and McLaren 1974). The *t* haplotype enhances sperm transport after insemination in vivo (Tessler and Olds-Clarke 1981) and influences the rate of egg penetration after insemination in vitro (Olds-Clarke and Carey 1978; Johnson et al. 1995; Redkar et al. 2000). Furthermore, in vitro fertilization experiments have shown nonrandom fertilization of eggs by sperm of different MHC types (Wedekind et al. 1996; Rülicke et al. 1998), and studies have shown fertilization and pregnancy failures (abortions) when the mating pair shares MHC alleles (Ober et al. 1992; Ho et al. 1994; Apanius et al. 1997; Rülicke et al. 1998). A paternity bias against related sperm in polyandrous kin/non kin matings in house mice is also consistent with avoidance of familial MHC (Firman and Simmons 2008b). Egg-sperm interactions are thought to produce mainly weak fertilization bias, with an effect size of 5% (Rülicke et al. 1998), in line with the 11% fertilization bias we observed in this study between *t* and + sperm from the same male. An alternative hypothesis, the sperm selection hypothesis (Ziegler et al. 2002), proposes an interaction between MHC and chemoreceptors that would result in sperm-egg interactions consistent with MHC-based fertilization bias (Ziegler et al. 2005, 2010).

MHC may also play a role in precopulatory sexual selection. Differences in MHC influence urinary odors in house mice (Carroll et al. 2002; Wilse et al. 2006; Kwak et al. 2009), which are readily detected (Yamazaki et al. 1990; Carroll et al. 2002; Kwak et al. 2009). Olfactory receptors can detect small MHC peptide ligands, in the vomeronasal organ and the main olfactory epithelium (Leinders-Zufall et al. 2004; Boehm and Zufall 2006). Volatile compounds in urine have been related to the MHC (reviewed in Kavaliers et al. 2005 and Kwak et al. 2010) and urine chemistry differs between +/*t* and +/+ males (Drickamer and Lenington 1987; Jemiolo et al. 1991). Female and male house mice have been reported to use odor cues to differentiate between +/*t* and +/+ in Y-maze choice tests (Egid and Lenington 1985; Lenington and Egid 1985). There is evidence for MHC-dependent mate choice in several species including house mice (Roberts 2009; Penn and Musolf 2012), but not every study finds such evidence (Milinski 2006; Penn and Musolf 2012). Where effects are found, they are typically weak (Milinski 2006). Thus, *t*-linked MHC could function as an anti-“green-beard” gene, a marker that individuals who bear it can use to recognize and avoid conspecifics that also bear it, in contrast to a green-beard gene (Dawkins 1976) for an altruistic trait (or kin recognition). However, Lenington and Egid (1985) and Lenington et al. (1988) proposed a compatibility and choice allele system within the *t* haplotype, as they found that females carrying a *t* haplotype recombinant for the distal portion of the fourth inversion, but which retained MHC, no longer showed a mate preference. Recent studies of MHC-linked olfactory receptor genes have shown that they may differ between mouse strains, and coduplicate with MHC loci (Amadou et al. 2003). As the *t* haplotype contains unique MHC alleles, it may also contain unique odor receptor genes. Physical linkage between MHC loci and odor receptor loci on a section of chromosome 17 protected from recombination could allow the evolution of a signal – receiver system as conceived by Lenington and Egid (1985) and Lenington et al. (1988). Olfactory receptor genes are candidates for such a choice allele. Thus, the MHC and its linked polymorphic olfactory receptor genes could provide a potential signal and recognition/mate choice system for the *t* haplotype, both for the individual and for sperm.

An alternative is that the fertilization bias is aimed at the MHC itself, to reduce homozygosity at MHC loci of the offspring. MHC heterozygosity enhances resistance to most infectious agents (reviewed in Penn et al. 2002 and Oliver et al. 2009, but see Ilmonen et al. 2007), increases host survival (Penn et al. 2002), and enhances reproductive success in wild mice (Thoß et al. 2011). MHC homozygosity can be correlated with close inbreeding (Roberts et al. 2006) and inbreeding depression is the most dramatic example of the importance of heterozygosity (Meagher et al. 2000; Keller and Waller 2002). In our sample excluding founder individuals, we found heterozygote excess for +/*t* at MHC and a nonsignificant deficit in +/+, with no difference in neutral microsatellites. A similar excess of heterozygotes at the MHC in +/*t* mice, and a heterozygote deficit in +/+ mice, was detected in an Israeli population, where the *t* haplotype was found at high frequency (Ben-Shlomo et al. 2007).

Individuals carrying the *t* haplotype were always heterozygous for MHC alleles, including the *t*-specific allele which +/+ never carry, and may therefore make attractive mating partners for +/+ individuals. The influence of MHC polymorphism on *t* frequencies has yet to be investigated. Mate choice for partners with different alleles at the MHC would result in a mating pattern whereby fertilizations between +/*t* males and +/*t* females or *t* sperm and *t* eggs are reduced, as they share MHC alleles, but fertilizations between +/*t* and +/+ individuals are not. The higher the frequency of *t* heterozygotes among the breeding population, the stronger should be the effect of discriminatory mate choice in reducing the frequency of transmission of the *t* haplotype to the next generation. Typically, wild mice populations have low *t* frequencies (Ardlie and Silver 1996; Huang et al. 2001), and in such cases one might find no difference in mate choice between +/*t* and +/+ females.

## Summary

In this study, we have shown that genetic incompatibility at the *t* haplotype imposes a high cost in terms of embryonic survival. In a population of wild house mice, we have found a weak, but significant, bias in mate choice, which reduces instances of *t*-associated genetic incompatibility. In the laboratory, we have documented a significant fertilization bias which reduces genetic incompatibility when +/*t* females mate with a +/*t* male. We have shown that a unique MHC allele is associated with the +/*t* haplotype in our study population. Tight linkage between the *t* haplotype and the MHC could be the key to mate choice bias in this system.

## Supporting Information

**Table S1.** Litter sizes and placental scar counts at birth resulting from different mating crosses.
