# Supplementary material for: Mate choice for genetic compatibility in the house mouse
Source: Ecol Evol. 2013 Mar 20;3(5):1231–47. doi: 10.1002/ece3.534 (PMC3678478; doi:10.1002/ece3.534)
Supplement: Supplementary file 1 [file ece30003-1231-SD1.docx]

Supplementary Table 1. Litter sizes and placental scar counts at birth resulting from different mating crosses. Total scars indicate the total number of implanted embryos, red scars reflect the numbers of these embryos surviving to birth, and yellow scars indicate the number of these embryos which died prenatally.

| Mating cross  (female X male) | N | Mean ± SE of birth litter size | Mean ± SE of total scars | Mean ± SE of red scars | Mean ± SE of yellow scars |
| --- | --- | --- | --- | --- | --- |
| Exp. 1 |  |  |  |  |  |
| *+/t* X +/*t* | 11 | 3.45 ± 0.46 |  |  |  |
| *+/t* X +/+ | 12 | 5.33 ± 0.83 |  |  |  |
| +/+ X +/*t* | 14 | 5.86 ± 0.50 |  |  |  |
| +/+ X +/+ | 16 | 5.75 ± 0.30 |  |  |  |
| Exp. 2 |  |  |  |  |  |
| +/*t* X +/*t* | 22 | 3.95 ± 0.39 | 7.27 ± 0.26 | 4.18 ± 0.42 | 3.09 ± 0.37 |
| +/*t* X +/+ | 21 | 7.00 ± 0.34 | 7.67 ± 0.28 | 7.00 ± 0.35 | 0.67 ± 0.29 |
| +/+ X +/*t* | 19 | 6.21 ± 0.46 | 7.47 ± 0.32 | 6.63 ± 0.38 | 0.84 ± 0.28 |
| +/+ X +/+ | 12 | 7.25 ± 0.37 | 8.17 ± 0.30 | 7.75 ± 0.31 | 0.42 ± 0.15 |
| Combined |  |  |  |  |  |
| +/*t*  X +/*t* | 33 | 3.78 ± 0.30 |  |  |  |
| +/*t*  X +/+ | 33 | 6.39 ± 0.39 |  |  |  |
| +/+ X +/*t* | 33 | 6.06 ± 0.34 |  |  |  |
| +/+ X +/+ | 28 | 6.39 ± 0.27 |  |  |  |
